# Mass flow and consumption calculations of pharmaceuticals in sewage treatment plant with emphasis on the fate and risk quotient assessment

**DOI:** 10.1038/s41598-023-30477-3

**Published:** 2023-03-01

**Authors:** Mohamed I. Badawy, Fatma A. El-Gohary, Mahmoud S. Abdel-Wahed, Tarek A. Gad-Allah, Mohamed E. M. Ali

**Affiliations:** Water Pollution Research Department, National Research Centre, Dokki, P.O. 12622, Giza, Egypt

**Keywords:** Environmental sciences, Risk factors, Chemistry

## Abstract

In Egypt, pharmaceuticals consumption increased dramatically owing to the population growth and the unrestricted sale manner. Accordingly, the occurrence and fate of nine common pharmaceutical active compounds (PhACs) were scrutinized at a sewage treatment plant (STP) in Giza, Egypt. The levels of these PhACs were assessed in different the wastewater treatment stages and dewatered sludge phase using high-performance liquid chromatography coupled with photodiode arrays detector. The average concentrations of the total PhACs detected in influent, primary sedimentation effluent (PSE) and final effluent (FE) were 227, 155 and 89 µg L^−1^, respectively. The overall removal efficiency of the individual PhACs ranged from 18 to 72% removal. The occurrence trend revealed that biodegradation and adsorption are the concurrently removal mechanisms of the studied PhACs. The overall consumption per day in West of Greater Cairo was estimated based on influent concentration of STP. Sulfamethoxazole, paracetamol and diclofenac were detected with the highest levels in the influent of STP, PSE and FE as well as in the dewatered sludge. Furthermore, the high concentrations of these compounds in the sludge confirm the adsorption pathway removal of theses PhACs. The risk quotient (RQ) assessment for the detected PhACs in FE is greatly higher than the predicted non-effect concentration (PNEC). Conclusively, the FE of STP is considered a risky source for PhACs in adjacent surface water.

## Introduction

Pharmaceutical active compounds (PhACs) are considered emerging risky pollutants to water resources. Among the various available antibiotics and anti-inflammatory drugs are used in both human and veterinary medicine. In consequence of progressively repeated medication prescriptions and pharmaceuticals consuming, PhACs and their byproducts have been identified in nearly all ecological parts^1,2^. Specifically, these emerging environmental contaminants have been detected globally in the discharge of sewage treatment plant (STP) and water resources at small levels, ranging from ppt to ppb according to their stability, biodegradation, physical–chemical properties and efficacy of the STP treatment^3^.

Existing remediation technologies in STPs are designed to remove particulate matters, and biodegradable organic matters, as well as nitrogen and phosphorus, but not pharmaceutical compounds^3,4^. Therefore, pharmaceuticals that are existed in domestic wastewater, are not biologically destructed in STPs; then, they are released to the surface water resources or adsorbed onto the sludge. Followed by, soil and water contaminated by these PhACs are infiltering the groundwater sources upon using the sludge as fertilizer.

Lately, overpowering concern about the existence of PhACs as persistent pollutants in the environment is noticed due to their impending harmful effects on aquatic environment and earthly wildlife^5–8^. Significant fractions of original PhACs are released as unmetabolized or as byproducts, via urine and feces of human body or veterinary, and are discharged into the sewerage networks, which is the chief entrance pathway of PhACs. The existences of residual PhACs in STP effluents have been determined in different countries. On other hand, The STPs efficiency are decreased due to the PhACs existence, which causes undesirable impacts on microorganisms that are responsible for the biological degradation of the organic materials^9–11^. The attention for emerging contaminants existence has increased because of their harmful influences of non-target living organisms. The occurrence of antibiotics could result in developing bacteria with antibiotic resistance genes; thus, their occurrence and fate have great concern of environmentalists. In addition, toxicity studies have highlighted the potential toxic effects of hospital effluents entering the aquatic environment^12–14^ and drug resistant bacteria have also been observed where hospital effluents are present^15–18^ Herein, the objectives of this work are to identify and quantify the levels of residual PhACs in the different stages of STP. The fate, mass balance and personal contribution for these pollutants have also been assessed. Additionally, risk quotient (RQ) assessment for the detected PhACs in final effluent is measured for the first time in Egypt.

## Materials and methods

### Description of the study sites

#### Zenin Sewage treatment plant (STP)

**Figure 1:**
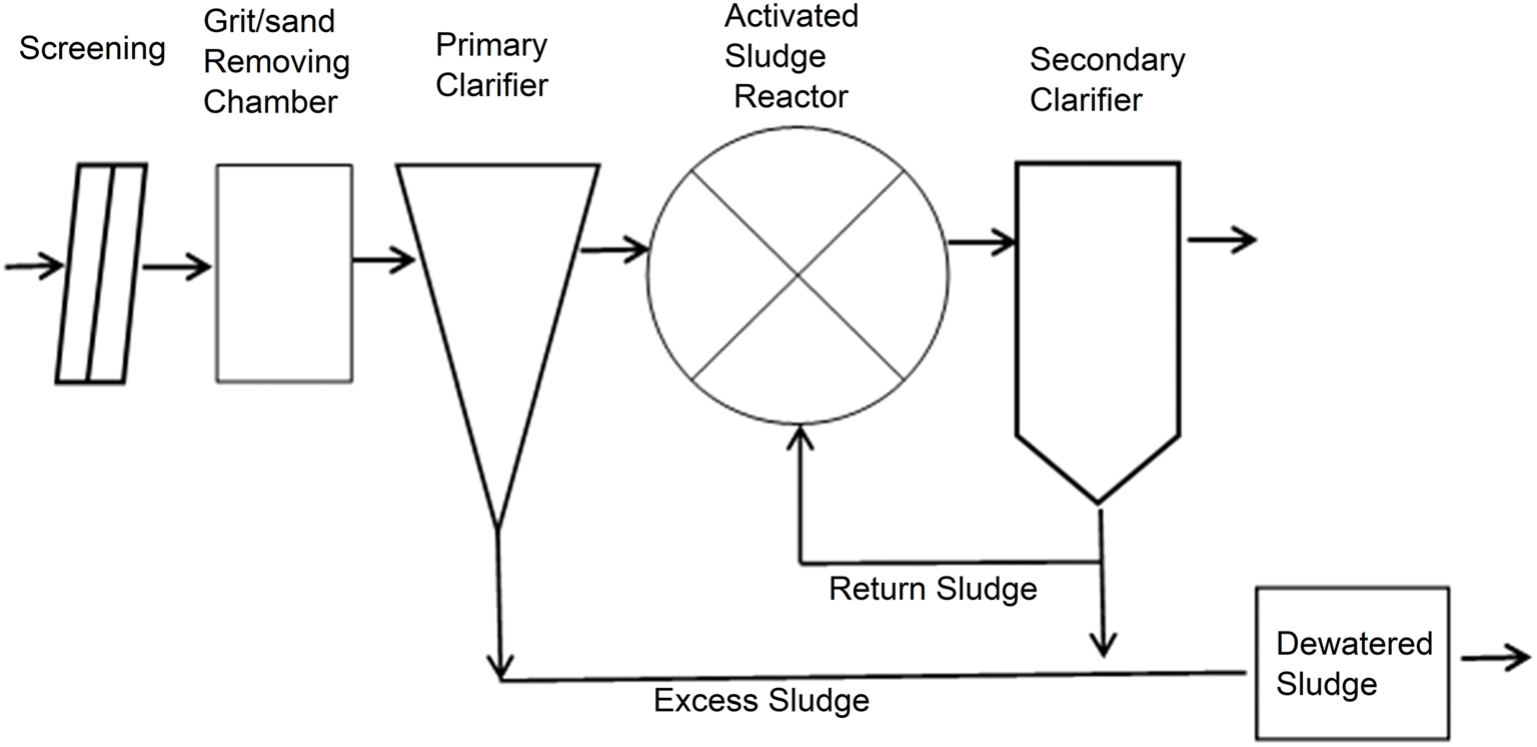
Process flow diagram for Zenin STP.

Zenin STP is located in Giza at the west of Greater Cairo. The plant has a daily capacity of 400,000 m^3^ and serving ca 2.5 million people. This municipal wastewater treatment plant includes a primary treatment step followed by an aerobic activated sludge for treating the combined wastewater as shown in Fig. 1. The plant consists of three identical separate modules. The treated effluent is discharged into Nahia drain reaching River Nile Rosetta branch via El-Rahawy Drain. Sludge treatment activity is performed at Zenin STP via thickening and drying into lagoons in the desert along Alexandria-Cairo Desert Road.

### Sampling, extraction and analysis

Different wastewater samples were collected from the STP stages (influent, primary sedimentation effluent (PSE), final effluent (FE)) for 12 h daily, where 500 mL of each stream (influent, PSE and FE) was collected every hour and then mixed to form the composite samples. The samples were placed in an ice-box, and then, were transferred immediately to the laboratories of the Water Pollution Research Department for preparation and analysis. Sludge samples were collected from the sludge holding tank for dewatered sludge from different location in the tank.

For quantification of PhACs in the wastewater, two hundred and fifty millilitres of the sample was filtered using 0.45 μm Nylon membrane filter (Phenomenex, Spain), then passed through C_18_ SPE cartridge. Prior application of the sample, C_18_ SPE cartridge with 500 mg/5 mL (Phenomenex, Spain) was conditioned by adding 5 mL distilled water, then 5 mL methanol, and finally 5 mL distilled water. Adsorbed PhACs were eluted from the SPE cartridge by acetonitrile. Then, the extracts were evaporated to dryness and re-dissolved in 2 mL methanol. The concentrated extracts were filtered using PTFE filter membrane prior to injection. Finally, 20 μL of the extract was injected into a high performance liquid chromatography (HPLC) equipped with photodiode array detector with wavelength range 190–900 nm (Agilent 1260 HPLC, USA). The used analytical column was Zorbax SB-C18 (4.6 mm i.d. × 250 mm, 5 μm). Oven temperature was kept at 35 °C during the analysis. Isocratic elution at a flow rate of 1 mL min^−1^ was carried out using the two eluents; Solution A was 0.017 M phosphate buffer (40%), while, solution B was acetonitrile (60%).

For quantification and analysis of PhACs in dewatered sludge, liquid–solid extraction procedure was applied using soxhlet technique; in which, 20 g of the dried sludge was extracted using dichloromethane-methanol (v:v, 50:50) as solvent. Then, the extract was evaporated to dryness and re-dissolved in 2 mL methanol for chromatography analysis.

### Quality control

The quality control program was carried out. This program includes the following:

1. Blank was run with each set of analysis.
2. Quantification of the studied PhACs was carried out using external standards with coefficient for calibration curves higher than 0.99.
3. The calibration program was verified on each working day by measuring one or more standards.
4. A random sample was run in triplicate. Laboratory control sample was analyzed with each series of samples (10 samples).
5. Q-chart was used and two values of ± 2 standard deviations are the lower and upper limits.
6. The method recovery for the PhACs was determined by spiking 200 mL ultrapure water with known concentration of the studied PhACs and ciprofloxacin as surrogate standard. The detected recoveries of the studied PhACs ranged from 83 to 103% and also, the recovery for ciprofloxacin was 93%.

### Calculation of removal efficiency

The performance of the wastewater treatment units, in terms of PhACs removal, was calculated using the difference in the concentration of the targeted compounds in the influent and the effluent of the relevant units. Total removal performance of the studied STP was calculated based on the detected concentrations of PhACs in the influent (raw wastewater) and the effluent (discharged wastewater) of the STPs.

Accordingly, the removal percentage of each compound (R %) from the influents at the different treatment units can be calculated based on the following equation:

1 $$R \%=\left(1-\left(\frac{{M}_{Effluent}}{{M}_{Influent}}\right)\right) \times 100$$

### Mass balance analysis

The average mass flow of each target compound was calculated as follow:

2 $$M_{aqueous}\left(\frac{g}{d}\right)=Q_{wastewater} \times C_{aqueous}\times 10^{-3}$$

3 $$M_{sludge}\left(\frac{g}{d}\right)=Q_{sludge} \times C_{sludge}$$

where, M_aqueous_ and M_sludge_ (g day^−1^) are the mass flows of the PhACs estimated in aqueous phase and the sludge, respectively, C_aqueous_ (µg L^−1^) and C_sludge_ (mg kg^−1^) are the determined concentrations in the aqueous phase and the sludge, respectively, and Q _wastewater_ (m^3^ day^−1^, where the flow rate of wastewater is 400,000 m^3^ day^−1^) and Q_sludge_ (ton day^−1^) are the flow rates of the wastewater and the production rate of the sludge (i.e. dewater sludge flow rate is 172 m^3^ day^−1^), respectively. The removal efficiency (R %) of the investigated PhACs during the wastewater treatment was calculated according to Eq. (1):

### Calculation of the pharmaceutical consumption

In this study, the investigated STP was considered representative for the general situation in west of Greater Cairo because the plant treats over 30% of the municipal wastewater in Giza Governorate located in Greater Cairo. The daily use of PhACs was back-estimated based on their concentrations in the influent of the STP according to the following formula:

4 $$U=\frac{{C}_{influent}\times {Q}_{wastewatr}\times {10}^{-3}}{0.5}$$

where, U represents the back-estimated usage of the target PhACs (kg year^−1^), C_influent_ (µg L^−1^), and Q (m^3^ day^−1^) refer to their previous definitions in Eqs. (2) and (3); 0.5 is a factor representing the ratio of the disposed drugs in wastewater to the drug sales that is according to phramakinetic for the most studied pollutants^19,20^.

### Environmental quotient risk assessment

The level of each compound was compared to the acute and chronic quality standards proposed by the Swiss Ecotox Centre. The levels of pharmaceuticals in the surface water and the effluent were compared to the maximum allowable concentration (MAC), which corresponds to the acute quality standards, and to the annual average concentration (AAC), corresponding to the chronic quality standards. The chronic quality standard is particularly relevant to the assessment of the impact of long-term pollution on the aquatic organisms. Furthermore, the potential risk of each pharmaceutical was assessed by calculating its risk quotient (RQ) defined as the ratio between the maximum measured environmental concentration and the predicted no-effect concentration (PNEC)^21^. PNEC is the concentration below which no adverse effects of exposure in an ecosystem are measured. It is calculated by applying an assessment factor (10 or 1000) on the lowest ecotoxicological values reported in the literature such as the no-observed-effect concentration (NOEC) or the concentration that causes adverse effects on 50% of the test organisms (EC_50_), respectively, for the most sensitive species assayed.

Risk quotient (RQ), which is the ratio between the measured concentration of each compound and their corresponding PNEC, was assessed for the final effluent to estimate the ecotoxicological potential of the studied nine PhACs in the wastewater discharged from the STP according to the following equation^22^.

6 $$RQ= \frac{{C}_{ FE}}{PNEC}$$

where, C_FE_ is the detected individual PhACs concentration in the final effluent.

## Results and discussion

### Occurrence and fate of the PhACs through the treatment train of Zenin STP

**Figure 2:**
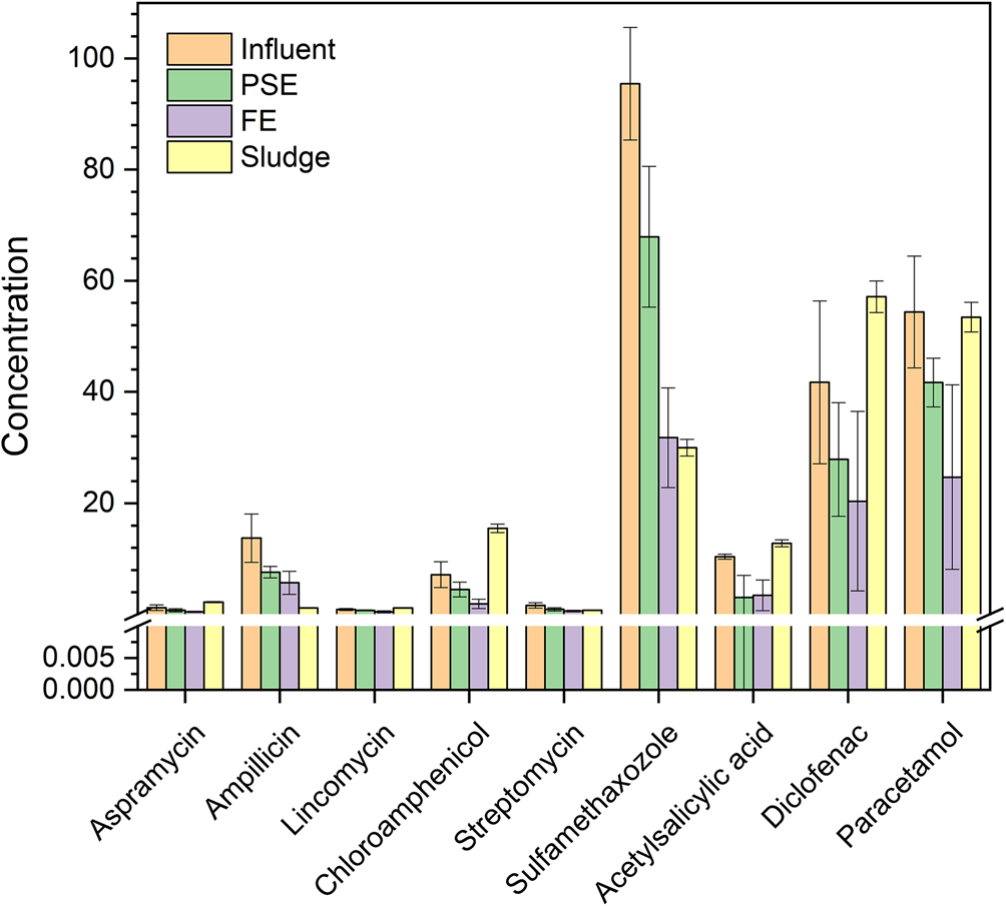
Existence of the different PhACs in the wastewater samples during the different stages of Zenin STP and the distribution of PhACs in the sludge (i.e. concentration unit for the liquid phase is µg L^−1^ and concentration unit for the sludge is mg kg^−1^).

The average influent flow in the STP during the sampling periods was 400,000 m^3^ day^−1^. While after the treatment, the daily production of dried sludge was approximately 116 ton day^−1^. In this study, nine PhACs pollutants were analyzed in the influent, PSE, FE of STP that located in the west of Greater Cairo. The average concentrations of the studied individual PhACs in the wastewater at the various stages of the treatment are summarized in Fig. 2. Sulfamethoxazole (85.36 and 105.64 µg L^−1^), paracetamol (42.74 and 65.45 µg L^−1^), diclofenac (26.48 and 55.76 µg L^−1^), and ampicillin (10.04 and 18.53 µg L^−1^) displayed the highest levels in the influent of STP. The analogue values for the PhACs in the PSE are sulfamethoxazole (58.34 and 82.28 µg L^−1^), paracetamol (36.72 and 45.12 µg L^−1^), diclofenac (17.91 and 38.28 µg L^−1^), and ampicillin (8.18 and 12.57 µg L^−1^). In the FE of STP, the most dominant PhACs are sulfamethoxazole, paracetamol, diclofenac, and ampicillin; where, the average levels of sulfamethoxazole, paracetamol, diclofenac, and ampicillin are amounted at 31.76, 24.68, 20.35, and 5.71 µg L^−1^, respectively. The results revealed that sulfamethoxazole, and diclofenac are much higher than that previously reported in STPs in China^21^ and France^22,23^. Diclofenac, paracetamol and sulfamethazole showed higher levels in the dewatered sludge than that previously reported in other regions^22,23^.

### Dominance and removal pathway of PhACs through the treatment train of Zenin STP

**Figure 3:**
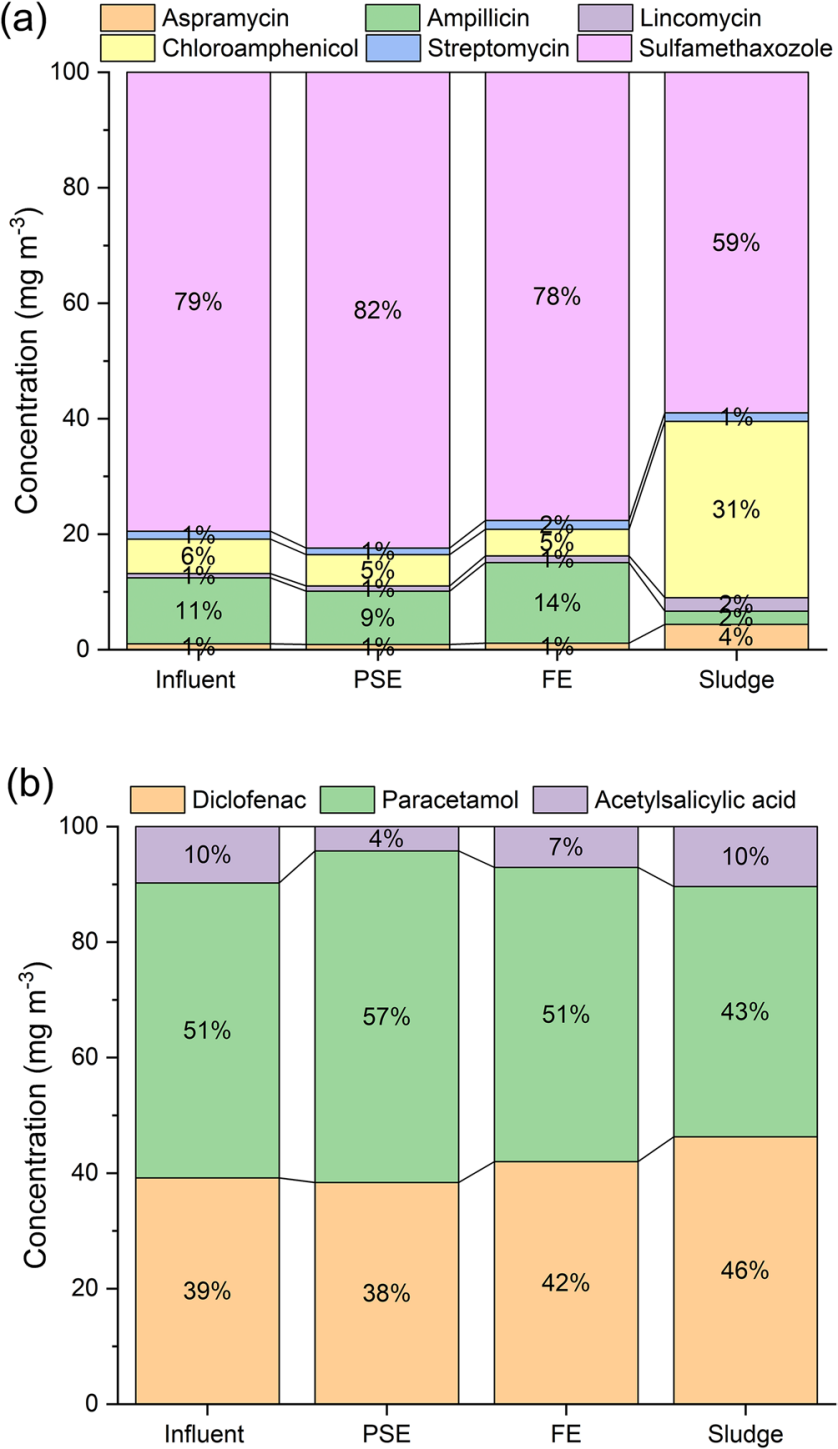
Distribution patterns of the pharmaceutical compounds in the different stages of STP.

Figure 3 depicts the distribution trends of PhACs in the different stages of STP; Influent, PSE, FE and sludge. The studied antibiotics (Fig. 3a) pattern indicates that sulfamethazole dominance represented 79.5% of the total antibiotics amount in the influent, 82.4% in the PSE and 77.6% in the FE; while, it amounted by 59% in the sludge. The trend of anti-inflammatory PhACs in Fig. 3b showed the following order paracetamol > diclofenac >  > acetylsalicylic acid. This indicates the higher consumption pattern of sulfamethazole, paracetamol and diclofenac as medications. The presence trends of the target PhACs indicate their participation into the different stream of STP and reflect the usage pattern of the PhACs in the service area to a definite level.

**Figure 4:**
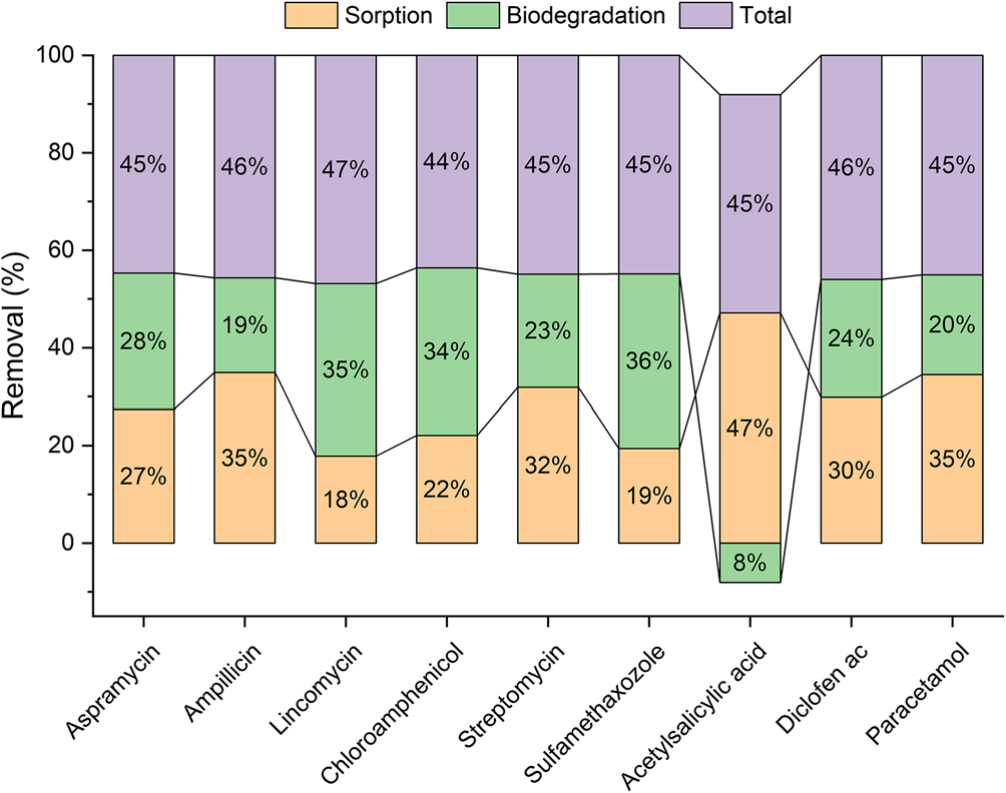
Removal percentages of pharmaceutical compounds in different stages of STP.

Additionally, different removal pathways of the PhACs were assessed. Variations in the removal % of PhACs in the STP are presented in Fig. 4. It was found that the adsorption and the biodegradation are the concurrent removal mechanisms of most of the studied PhACs. Additionally, the results reveal that acetylsalicylic acid was removed in particular via adsorption on the particulate matter of sludge with an efficiency of 45%. The other anti-inflammatory PhACs were partially removed via the adsorption-assisted removal mechanism. Meanwhile, the removal mechanism for the antibiotics is a combinational pathway of adsorption–biodegradation. The recorded adsorption and biodegradation rates were 33% and 26% for diclofenac, and 41.66 and 24.68% for paracetamol, respectively, revealing their lower removing by the biodegradation process. These findings are inconsistence with the previous data^23,24^. Removal rate of  the studied antibiotics via adsorption ranged from 18 to 35%. Meanwhile, biodegradation rate of the studied antibiotics varied from 19 to 36% indicating low biodegradation transformation of the antibiotic by the activated sludge bacteria.

### Mass balance of the PhACs in the different stages of STP

**Figure 5:**
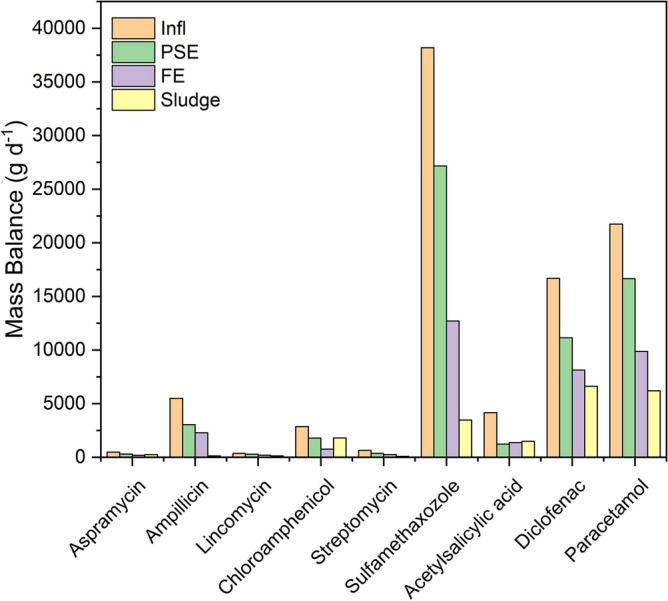
Mass balance of the PhACs along different stages of STP.

**Figure 6:**
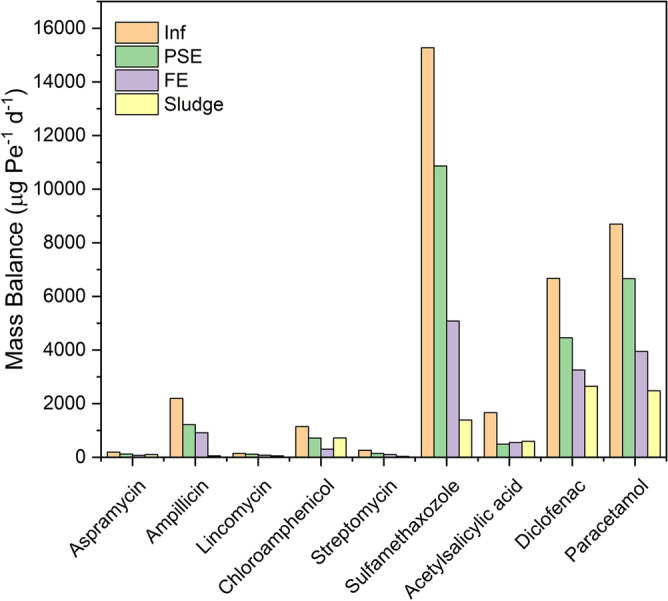
Personal mass load of the PhACs along the different stages of STP.

Mass flow and mass balance of the individual PhACs were investigated to evaluate their potential in the STP effluent on the receiving surface water bodies and the data are shown in Fig. 5. The influent mass load for all target PhACs was 90,616 g day^−1^. Mass loads in the PSE and the final effluent of all the studied PhACs were 61,976 g day^−1^ and 35,748 g day^−1^, respectively. The mass loads of the individual PhACs varied from 484 to 38,184 g day^−1^ for aspramycin and sulfametazole, respectively, in the influent, and from 184 to 12,704 g day^−1^ in the final effluent of STP. The adsorbed amounts were remarkable for paracetamol and diclofenac. The mass load of the selected PhACs in the dewatered sludge (without further digestion) was 20,193 g day^−1^. Diclofenac and paracetamol were the predominant residual PhACs in the dried sludge. Figure 6 showed the personal load for different PhACs, the results showed the same of mass balance. The personal median mass load (C_pe_, × 10^3^ µg day^−1^ Pe^−1^) of the individual PhACs was estimated according to multiplying the concentration in the sewage (C_i_, µg L^−1^) and the average treated flow rate (Q, m^3^. day^−1^) during the sampling period and normalizing this value to the population served by the corresponding STP. The equation is expressed as following;

5 $$C_{pe} = C_{inf} \times \, Q_{wastewater} \times 1000/served \, population \, number$$

The median values are shown in Fig. 5. The mass load of the target PhACs in influent revealed the input into STP and indicated the use pattern of PhACs in the served region to a definite magnitude. By contrast, the PhACs mass load in the effluent is used to estimating the risk of PhACs to the water resources. The total average personal mass flow for the studied PhACs was found to be 70.2 × 10^3^ µg day^−1^ Pe^−1^ and 51.83 × 10^3^ µg day^−1^ Pe^−1^ for the influent of WTP and the effluent to the receiving water. The daily mass flow per person for the analgesic and the anti-inflammatory drugs is 50.8 × 10^3^ µg day^−1^ Pe^−1^ for the influent of WTP, which is 2.6 time higher than the daily mass flow per person of the total antibiotic (i.e. 19.3 × 10^3^ µg day^−1^ Pe^−1^), which shall be endorsed to the fame and common use of these drugs. However, daily flow mass per capita was detected in the current served region is many times higher than that detected in Spain (7485 day^−1^ Pe^−1^), the United States (15,440 µg day^−1^ Pe^−1^), China (4950 µg day^−1^ Pe^−1^) and France (4774.7 µg day^−1^ Pe^−1^)^21,23,25,26^. Meanwhile, the calculated daily personal mass flow of the analgesics and the anti-inflammatory drugs and the antibiotics into the receiving water are 36.79 × 10^3^ µg day^−1^ Pe^−1^ and 14.04 × 10^3^ µg day^−1^ Pe^−1^, respectively. These discharged levels are much higher than those detected in other regions^21,23,25,26^. The mass flow levels of the two other analgesics, namely, paracetamol and diclofenac are found to be 8690 and 6670 µg day^−1^ Pe^−1^ in the influent, respectively. These values are many times higher than those determined in the industrialized countries such as USA (IBP: 8257 µg day^−1^ Pe^−1^; DCF: 103 µg day^−1^ Pe^−1^)^27^, Sweden (IBP: 920 µg day^−1^ Pe^−1^; DCF: 31 µg day^−1^ Pe^−1^)^28^, Spain (IBP: 1983 µg day^−1^ Pe^−1^; DCF: 72 µg day^−1^ Pe^−1^)^26^, and Greece (IBP: 165 µg day^−1^ Pe^−1^; DCF: 311 µg day^−1^ Pe^−1^). As well, the mass flow levels of common antibiotics, namely, sulfamethazole and ampicillin are found to be 15,280 and 2198.4 µg day^−1^ Pe^−1^ in the influent, respectively, that are higher in comparable with the previously investigated in other regions^26,28–30^. Sulfamethazole, diclofenac and paracetamol had the highest average daily mass load per person in the effluent as shown in Fig. 6. To our knowledge, the calculated daily mass load per person for aspramycin, lincomycin, streptomycin and chloramphenicol have not been previously investigated in other countries in STP streams. Finally, the PhACs detected in the current study were compared with the available data in the previous literature as presented in Table 1. The PhACs concentrations detected in this study is slightly higher than data shown lately in the literature. So that, these compounds have possible environmental threats.

**Table 1:** International comparison for PhACs levels with current study.

PhACs	PhACs levels in influent (ng L^−1^)	PhACs levels in Effluent (ng L^−1^)	References
Sulfamethoxazole	–	< 1000	^39^
Sulfapyridine	251.7	99.9	^40^
Sulfamethazine	23.7	11.0	
Sulfadiazine	720.2	433.4	
Sulfamethoxazole	161.8	75.1	
Ciprofloxacin	862.7	543.4	
Ofloxacin	845.9	510.8	
Erythromycin	785.2	541.2	
Acetaminophen	145,250.3	5235.3	
Metoprolol	92.1	62.5	
Risperidone	244.8	13.2	
Diclofenac	–	965–2476	^22^
Sulfamethoxazole	–	655–1380	
Carbamazepine	–	566–1007	
Sulfadiazine	202.8–257.8	117.8–174.8	^21^
Sulfamethazine	129–174.4	31.7–47.2	
Sulfamethoxazole	2460.4–3180	1060.3–1212.2	
Trimethoprim	51.9–98.8	37.9–75.5	
Ofloxacin	276.7–401.5	43.0–82.9	
Norfloxacin	186.3–225.1	25.5–34.2	
Moxifloxacin	27.6	5.7–7.7	
Erythromycin	238.6–275.4	135.9–174.0	
Roxithromycin	359.7–434.6	300.6–386.4	
Azithromycin	330.27–376.5	58–111.0	
Ibuprofen	243.8–296.5	11.9–16.3	
Diclofenac	6.01	2.9–3.7	
Acetaminophen	6813.5–7515.6	10	
Bezafibrate	98.75–140.13	50.2–92.3	
Clofibric acid	17.5–47.9	15.4–17.1	
Gemfibrozil	12.7–18.2	2.7–3.4	
Metoprolol	43.18–54.1	51.1–82.5	
Amlodipine	–	4.6–5.3	
Atorvastatin	1.0–1.9	0.6	
Simvastatin	101.4–133.7	30.2	
Carbamazepine	9.8–20.1	13.6–21.0	
Acetaminophen	22,600–96,700	13–172	^23^
Atenolol	3560–26,500	893–9320	
Carbamazepine	51–937	5–357	
Codeine	270–2110	137–518	
Diazepam	420	30	
Doxepin	92–1020	28–299	
Gemfibrozil	648	105	
Ketoprofen	149–6560	10–176	
Metoprolol	277–2760	121–1750	
Naproxen	457–4740	58–238	
Oxazepam	154–2020	5–1130	
Acetyl salicylic acid	2360–25,500	7–423	
Tramadol	1350–9860	72–1190	
Diclofenac	63–1190	43–1380	
Ibuprofen	1560–7280	6–284	
Sulfamethoxazole	85,360–105,640	31,760	This work
Paracetamol	42,740–65,450	24,680	
Diclofenac	26,480–55,760	20,350	
Ampicillin	10,040–18,530	5710	

### Consumption assessment

Based on the existed level of the selected PhACs and their pharmokinetics, the estimated calculations of the daily and annual consumption per person in the studied region are shown in Table 2. The obtained result illustrated that the most consumable PhACs are diclofenac, paracetamol and sulfamethazole owe to these values were compared with previously reported data in China^21^ (Yan et al., 2014), Spain^31,32^, Netherland^33^, Switzerland^34,35^, Germany^36^, Poland^37^. It was found that sulfamethazole, and diclofenac show an annual consumption per person higher than that recorded in the abovementioned regions. Meanwhile, paracetamol consumption showed lower value than that previously reported in different regions, China (11.82 g year^−1^ Pe^−1^)^21^, and Spain (31.05 g year^−1^ Pe^−1^)^31^. The consumption of antibiotics per capita has higher rate in west of greater Cairo compared with the developed countries by 18–95 orders of magnitude^21,31^. Generally, the annual per capita consumption of the non-steroid anti-inflammatories (diclofenac and acetyl salicylic acid) was higher many times than those reported in the developed countries^21^.

**Table 2:** Predicated back-estimated consumption of the studied PhACs in the studied region.

PhACs	Average personal consumption (mg day^−1^ Pe^−1^)	Estimated annual consumption (g year^−1^ Pe^−1^)
Aspramycin	0.77	0.277
Ampillicin	8.79	3.16
Lincomycin	0.57	2.05
Chloroamphenicol	4.58	1.66
Streptomycin	1.04	0.374
Sulfamethaxozole	61.09	22
Acetylsalicylic acid	6.66	2.4
Diclofenac	26.69	9.61
Paracetamol	34.79	12.52

### Risk quotient (RQ) of the final effluent

Risk quotient (RQ), which is the ratio between the measured concentration of each compound and their corresponding PNEC, was assessed for the final effluent to estimate the ecotoxicological potential of the studied nine PhACs in the wastewater discharged from the STP according to the following equation^38^.

6 $$RQ= \frac{{C}_{ FE}}{PNEC}$$

where, C_FE_ is the detected individual PhACs concentration in the final effluent.

**Figure 7:**
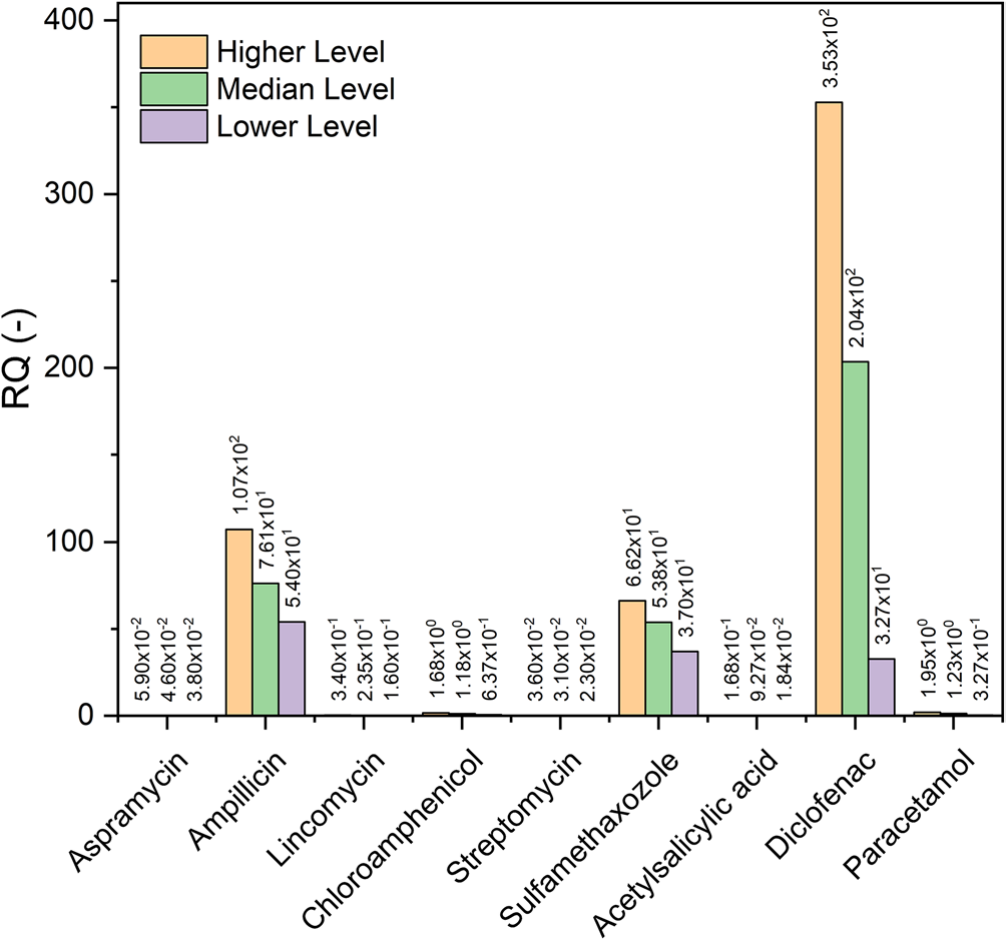
Risk quotients (RQs) of PhACs in STP effluents.

RQ of the FE showed that ampillicin, diclofenac, and sulfamethoxazole were the most critical PhACs and posed a medium and higher RQ, which, represents a pronounced environmental risk for the receiving surface water (see Fig. 7). It was found that RQ of diclofenac is 204 higher than that of PNEC. Additionally, the value of RQ for ampicillin is 76 higher than PNEC; respectively which results in increasing existence of antibiotic resistance bacteria in the surrounding environment. Other selected PhACs showed lower and non-risky value of RQ to receiving water. According to EC technical regulation documentation on RQ values from 0.01 to 0.1 are well-defined as low risk, 0.1 to 1 as medium risk and values > 1 as high risk (Communities, 1996)^39^.

Hitherto, international and local legislation has not provided limits for the concentrations of PhACs in the STP effluent. However, comparing the levels of PhACs in effluent showed that the above mentioned compounds surpass the long-term quality standard.

## Conclusion

In this work, the levels of nine PhACs contaminants were analysed along the wastewater treatment train of Zenin sewage treatment plant. It is worth mentioning that paracetamol, sulfamethazole and diclofenac showed relatively high concentrations in the STP influent and effluent. The most dominant PhACs in STP effluent were diclofenac, paracetamol, acetylsalicylic acid sulfamethazole and ampicillin. The removal rate for the antibiotics via adsorption ranged from 18 to 35%. Meanwhile, biodegradation rate of the studied antibiotics varied from 19 to 36%, indicating lower biodegradation competency by the activated sludge bacteria. The calculated daily mass load per person for aspramycin, lincomycin, streptomycin and chloramphenicol have not been previously investigated in other countries in STP streams. Finally, it was found that STP represents a major source for discharge of PhACs to the environment. The data obtained showed high mass flow levels for paracetamol and diclofenac in final effluent of STP. However, diclofenac, ampillicin, and sulfamethazole show risk quotient ten times higher than their corresponding PNEC values. The levels of aspramycin, streptomycin, and Lincomycin recorded the lower RQ to receiving water bodies. The results obtained revealed higher annual personel consumption for sulfamethazole, and diclofenac rather than the paracetamol consumption.

## Data Availability

All data generated or analyzed during this study are included in this published article.

## References

[1] Bouissou-Schurtz C (2014). Ecological risk assessment of the presence of pharmaceutical residues in a French national water survey. Regul. Toxicol. Pharmacol..

[2] Nieto-Juárez JI, Torres-Palma RA, Botero-Coy AM, Hernández F (2021). Pharmaceuticals and environmental risk assessment in municipal wastewater treatment plants and rivers from Peru. Environ. Int..

[3] Andreozzi R, Marotta R, Pinto G, Pollio A (2002). Carbamazepine in water: Persistence in the environment, ozonation treatment and preliminary assessment on algal toxicity. Water Res..

[4] Hai FI (2018). Carbamazepine as a possible anthropogenic marker in water: Occurrences, toxicological effects, regulations and removal by wastewater treatment technologies. Water.

[5] Bendz D, Paxéus NA, Ginn TR, Loge FJ (2005). Occurrence and fate of pharmaceutically active compounds in the environment, a case study: Höje River in Sweden. J. Hazard. Mater..

[6] Bertanza G, Collivignarelli C, Pedrazzani R (2001). The role of chemical oxidation in combined chemical-physical and biological processes: Experiences of industrial wastewater treatment. Water Sci. Technol..

[7] Martínez JL (2008). Antibiotics and antibiotic resistance genes in natural environments. Science.

[8] Li S (2021). Technologies towards antibiotic resistance genes (ARGs) removal from aquatic environment: A critical review. J. Hazard. Mater..

[9] Gómez MJ, Bueno MM, Lacorte S, Fernández-Alba AR, Agüera A (2007). Pilot survey monitoring pharmaceuticals and related compounds in a sewage treatment plant located on the Mediterranean coast. Chemosphere.

[10] Badawy MI, Wahaab RA, El-Kalliny A (2009). Fenton-biological treatment processes for the removal of some pharmaceuticals from industrial wastewater. J. Hazard. Mater..

[11] Xiong R (2022). Photodegradation of chloramphenicol in micro-polluted water using a circulatory thin-layer inclined plate reactor. Chemosphere.

[12] Hartmann A, Alder AC, Koller T, Widmer RM (1998). Identification of fluoroquinolone antibiotics as the main source of umuC genotoxicity in native hospital wastewater. Environ. Toxicol. Chem. Int. J..

[13] Emmanuel E, Perrodin Y, Keck G, Blanchard J-M, Vermande P (2005). Ecotoxicological risk assessment of hospital wastewater: A proposed framework for raw effluents discharging into urban sewer network. J. Hazard. Mater..

[14] Majumder A, Gupta AK, Ghosal PS, Varma M (2021). A review on hospital wastewater treatment: A special emphasis on occurrence and removal of pharmaceutically active compounds, resistant microorganisms, and SARS-CoV-2. J. Environ. Chem. Eng..

[15] Jung R, Fish D, Obritsch M, MacLaren R (2004). Surveillance of multi-drug resistant *Pseudomonas aeruginosa* in an urban tertiary-care teaching hospital. J. Hosp. Infect..

[16] Caplin JL, Hanlon GW, Taylor HD (2008). Presence of vancomycin and ampicillin-resistant *Enterococcus faecium* of epidemic clonal complex-17 in wastewaters from the south coast of England. Environ. Microbiol..

[17] Tuméo E (2008). Are antibiotic-resistant *Pseudomonas aeruginosa* isolated from hospitalised patients recovered in the hospital effluents?. Int. J. Hyg. Environ. Health.

[18] Mapipa Q, Digban T, Nnolim N, Nwodo U (2021). Antibiogram profile and virulence signatures of *Pseudomonas aeruginosa* isolates recovered from selected agrestic hospital effluents. Sci. Rep..

[19] Daughton CG, Ruhoy IS (2009). Environmental footprint of pharmaceuticals: The significance of factors beyond direct excretion to sewers. Environ. Toxicol. Chem..

[20] Zhou L-J (2013). Excretion masses and environmental occurrence of antibiotics in typical swine and dairy cattle farms in China. Sci. Total Environ..

[21] Yan Q (2014). Occurrence and fate of pharmaceutically active compounds in the largest municipal wastewater treatment plant in Southwest China: Mass balance analysis and consumption back-calculated model. Chemosphere.

[22] Chiffre A, Degiorgi F, Buleté A, Spinner L, Badot P-M (2016). Occurrence of pharmaceuticals in WWTP effluents and their impact in a karstic rural catchment of Eastern France. Environ. Sci. Pollut. Res..

[23] Thiebault T, Boussafir M, Le Milbeau C (2017). Occurrence and removal efficiency of pharmaceuticals in an urban wastewater treatment plant: Mass balance, fate and consumption assessment. J. Environ. Chem. Eng..

[24] Verlicchi P, AlAukidy M, Zambello E (2012). Occurrence of pharmaceutical compounds in urban wastewater: Removal, mass load and environmental risk after a secondary treatment—a review. Sci. Total Environ..

[25] Gracia-Lor E, Sancho JV, Serrano R, Hernández F (2012). Occurrence and removal of pharmaceuticals in wastewater treatment plants at the Spanish Mediterranean area of Valencia. Chemosphere.

[26] Karthikeyan K, Meyer MT (2006). Occurrence of antibiotics in wastewater treatment facilities in Wisconsin, USA. Sci. Total Environ..

[27] Yu Y, Liu Y, Wu L (2013). Sorption and degradation of pharmaceuticals and personal care products (PPCPs) in soils. Environ. Sci. Pollut. Res..

[28] Zorita S, Mårtensson L, Mathiasson L (2009). Occurrence and removal of pharmaceuticals in a municipal sewage treatment system in the south of Sweden. Sci. Total Environ..

[29] Castiglioni S (2006). Removal of pharmaceuticals in sewage treatment plants in Italy. Environ. Sci. Technol..

[30] Watkinson A, Murby E, Costanzo S (2007). Removal of antibiotics in conventional and advanced wastewater treatment: Implications for environmental discharge and wastewater recycling. Water Res..

[31] de García SO, Pinto GP, Encina PG, Mata RI (2013). Consumption and occurrence of pharmaceutical and personal care products in the aquatic environment in Spain. Sci. Total Environ..

[32] Carballa M, Omil F, Lema JM (2008). Comparison of predicted and measured concentrations of selected pharmaceuticals, fragrances and hormones in Spanish sewage. Chemosphere.

[33] Oosterhuis M, Sacher F, Ter Laak TL (2013). Prediction of concentration levels of metformin and other high consumption pharmaceuticals in wastewater and regional surface water based on sales data. Sci. Total Environ..

[34] ter Laak TL, van der Aa M, Houtman CJ, Stoks PG, van Wezel AP (2010). Relating environmental concentrations of pharmaceuticals to consumption: A mass balance approach for the river Rhine. Environ. Int..

[35] Göbel A, Thomsen A, McArdell CS, Joss A, Giger W (2005). Occurrence and sorption behavior of sulfonamides, macrolides, and trimethoprim in activated sludge treatment. Environ. Sci. Technol..

[36] Ternes T, Joss A (2006). Human Pharmaceuticals, Hormones and Fragrances—The Challenge of Micropollutants in Urban Water Management.

[37] Carballa M (2004). Behavior of pharmaceuticals, cosmetics and hormones in a sewage treatment plant. Water Res..

[38] U.S, F. D. A. Guidance for Industry-Environmental Assessment of Human Drug and Biologics Applications (U.S. FDA, CDER, CBER). http://www.fda.gov/cder/guidance/1730fnl.pdf (1998).

[39] Barber LB (2013). Persistence and potential effects of complex organic contaminant mixtures in wastewater-impacted streams. Environ. Sci. Technol..

[40] Semerjian L, Shanableh A, Semreen MH, Samarai M (2018). Human health risk assessment of pharmaceuticals in treated wastewater reused for non-potable applications in Sharjah, United Arab Emirates. Environ. Int..

